# Complete Genome Sequence of Clostridium botulinum CJ0611A1, a Type A(B) Isolate Associated with an International Outbreak of Botulism from Commercial Carrot Juice

**DOI:** 10.1128/MRA.01111-20

**Published:** 2021-01-07

**Authors:** Richard Harris, Forest Dussault, Annika Flint, John W. Austin, Kelly Weedmark

**Affiliations:** a Bureau of Microbial Hazards, Health Canada, Ottawa, Ontario, Canada; University of Arizona

## Abstract

We report the complete genome (3.9-Mb chromosome, 5.9-kb plasmid) of Clostridium botulinum CJ0611A1, a type A(B) strain isolated from carrot juice distributed in Canada and linked to an international 2006 foodborne botulism outbreak. This strain encodes a full-length *bont/A1* gene and a truncated *bont/B* gene.

## ANNOUNCEMENT

Clostridium botulinum is a Gram-positive, spore-forming, anaerobic bacterium capable of producing botulinum neurotoxin (BoNT), the causative agent of botulism. BoNTs fall into one of seven distinct serological types (A to G), each with several subtypes (1). In 2006, an international outbreak of botulism in Canada and the United States was traced to the consumption of commercial pasteurized carrot juice that contained BoNT type A (2, 3). Brand X carrot juice, associated with outbreaks in Georgia and Florida, and brand Y carrot juice, associated with an outbreak in Ontario, were produced by the same manufacturer in the same plant. C. botulinum isolated from carrot juice in Georgia (CDC51303) was found to carry the *bont/A1* gene, while a Florida isolate (CDC51348) contained a *bont/A* and an unexpressed *bont/B* gene (4–6). C. botulinum CJ0611A1 is a type A strain isolated from brand Y carrot juice recovered from a refrigerator shared by the two Ontario patients (3).

A single-colony pick from the original food specimen was grown on MT-EYE agar (1.5% McClung-Toabe agar [Difco, Tucker, GA], 5% egg yolk extract, and 5% yeast extract [Difco]) and then frozen at −80°C in multiple Microbank (Pro-Lab Diagnostics, Inc.) cryovials without further subculture. A bead from frozen stock was struck onto MT-EYE agar and incubated overnight at 30°C under anaerobic conditions. Cells from a single-colony TPGY (5% tryptone, 0.5% peptone, 0.4% glucose, 2% yeast extract, and 0.1% sodium thioglycolate) inoculate were collected after 24 h of anaerobic growth at 35°C in TPGY, and DNA was extracted using the Zymo Quick-DNA HMW MagBead kit following resuspension in Zymo DNA/RNA Shield. Eluted DNA was treated with RNase A (2 µl at 10 mg/ml, 30 min at 37°C) and purified using >1,500 bp solid-phase reversible immobilization (SPRI) selection (7). Illumina paired-end (2 × 300 bp) Nextera XT library sequencing was performed on a MiSeq instrument (v3 chemistry) according the manufacturer’s instructions (Illumina). Raw Illumina reads (863,750 reads, 301 bp average) were processed using Fastp (v 0.20.0) (8) to remove adapter and barcode sequences, correct mismatched bases in overlaps, and filter low-quality reads. MinION sequencing was performed using a PCR barcoding kit (SQK-PBK004) and SQK-LSK108 sequencing kit on a FLO-106 flow cell (Oxford Nanopore). Following Guppy base calling (Guppy GPU v 3.3.3+fa743ab), raw MinION reads (338,142 reads, 6,431 bp average) were quality control (QC) checked (Nanoplot v 1.29.0) (9), and reads of <10 kb were filtered out (Filtlong v 0.2.0; github.com/rrwick/Filtlong). The hybrid assembly (Unicycler v 0.4, normal mode) (10) was generated using quality-filtered Illumina (8,416,032 reads, 218 bp average) and MinION (74,388 reads, 16,934 bp average) reads, annotated with PGAP v 2020-07-09.build4716 (best-placed reference protein set, GeneMarkS-2) (github.com/ncbi/pgap), and analyzed with QUAST v 5.0.0 (github.com/ablab/quast). Clustal W v2.1 (11) was used to align *bont* genes and cassettes using references from reference 4.

The closed genome (321-fold coverage) has 28.28% GC content and comprises a circular chromosome (3,918,289 bp) and a plasmid (5,926 bp). A total of 3,450 complete protein-coding DNA sequences (CDSs), 93 pseudogenes, and 80 tRNAs are predicted. The CJ0611A1 genome contains a full-length chromosomal *bont/A1* gene (100% nucleotide identity with ATCC 3502 *bont/A1*, GenBank accession number AM412317) within an *ha^–^ orf^+^* cassette, as well as a truncated *bont/B* sequence ∼49 kb downstream in an *ha^+^ orf^–^* cassette. Based on these genomic data as well as toxin assays (4), CJ0611A1, like the Florida isolate (CDC51348), contains an unexpressed *bont/B* gene and is therefore a type A(B) strain (3).

### Data availability.

The CJ0611A1 genome sequence is available in NCBI (GenBank accession numbers CP059677.1 and CP059678.1); the reads have been deposited in the SRA (SRX8894092 and SRX8894093).
